# MiR‐126a‐5p limits the formation of abdominal aortic aneurysm in mice and decreases ADAMTS‐4 expression

**DOI:** 10.1111/jcmm.15422

**Published:** 2020-05-29

**Authors:** Lei Li, Wei Ma, Shuang Pan, Yongqi Li, Han Wang, Biao Wang, Raouf A. Khalil

**Affiliations:** ^1^ Department of Vascular Surgery The Second Affiliated Hospital of Dalian Medical University Dalian China; ^2^ Vascular Surgery Research Laboratories Division of Vascular and Endovascular Surgery Brigham and Women's Hospital Harvard Medical School Boston MA USA; ^3^ Department of Anatomy Dalian Medical University Dalian China; ^4^ Department of Physiology School of Basic Medicine Jinzhou Medical University Jinzhou China; ^5^ Graduate School of Comprehensive Human Sciences University of Tsukuba Tsukuba Japan; ^6^ Department of Vascular Surgery Dalian University Affiliated Xinhua Hospital Dalian China; ^7^ Department of Biochemistry and Molecular Biology School of Life Sciences China Medical University Shenyang China

**Keywords:** abdominal aortic aneurysm, ADAMTS‐4, ECM degradation, elastic fragment, MiR‐126a‐5p

## Abstract

Abdominal aortic aneurysm (AAA) is a serious vascular disease featured by inflammatory infiltration in aortic wall, aortic dilatation and extracellular matrix (ECM) degradation. Dysregulation of microRNAs (miRNAs) is implicated in AAA progress. By profiling miRNA expression in mouse AAA tissues and control aortas, we noted that miR‐126a‐5p was down‐regulated by 18‐fold in AAA samples, which was further validated with real‐time qPCR. This study was performed to investigate miR‐126a‐5p's role in AAA formation. In vivo, a 28‐d infusion of 1 μg/kg/min Angiotensin (Ang) II was used to induce AAA formation in Apoe^‐/‐^ mice. MiR‐126a‐5p (20 mg/kg; MIMAT0000137) or negative control (NC) agomirs were intravenously injected to mice on days 0, 7, 14 and 21 post‐Ang II infusion. Our data showed that miR‐126a‐5p overexpression significantly improved the survival and reduced aortic dilatation in Ang II‐infused mice. Elastic fragment and ECM degradation induced by Ang II were also ameliorated by miR‐126a‐5p. A strong up‐regulation of ADAM metallopeptidase with thrombospondin type 1 motif 4 (ADAMTS‐4), a secreted proteinase that regulates matrix degradation, was observed in smooth muscle cells (SMCs) of aortic tunica media, which was inhibited by miR‐126a‐5p. Dual‐luciferase results demonstrated ADAMTS‐4 as a new and valid target for miR‐126a‐5p. In vitro, human aortic SMCs (hASMCs) were stimulated by Ang II. Gain‐ and loss‐of‐function experiments further confirmed that miR‐126‐5p prevented Ang II‐induced ECM degradation, and reduced ADAMTS‐4 expression in hASMCs. In summary, our work demonstrates that miR‐126a‐5p limits experimental AAA formation and reduces ADAMTS‐4 expression in abdominal aortas.

## 1 INTRODUCTION

Abdominal aortic aneurysm (AAA) is a potential lethal vascular disease in the event of aortic rupture.^1^ Currently, therapies are limited to open surgical and less invasive endovascular repair.^2^ Effective drug‐based intervention for this disease is still lacking,^3^ highlighting an urgent need to better understand the underlying mechanisms involved in the formation and progression of AAA. The pathogenesis behind AAA is multifactorial, but it is well‐known that extracellular matrix (ECM) degradation and loss of vascular smooth muscle cells (VSMCs) occur, which ultimately contribute to the weakening and of remodelling the aortic wall.^4^, ^5^

Members of ADAM metallopeptidase with thrombospondin type 1 motifs (ADAMTSs) are secreted enzymes sharing a common multidomain structure, and participate in the degradation of ECM proteins during development, morphogenesis and tissue remodelling.^6^ ADAMTS‐4 is a secreted metalloproteinase which belongs to the ADAMTS family, and it cleaves various ECM proteins including brevican, versican and aggrecan in the vasculature.^7^ Increasing evidence has revealed an involvement of ADAMTS‐4 in the pathogenesis of aneurysmal diseases. Ren and co‐workers found that ADAMTS‐4 expression was much higher in aortic tissues of patients with thoracic aortic aneurysm and dissection (TAA/D) than in control aortas.^8^ This team further demonstrated that ADAMTS‐4 deficiency attenuated angiotensin II (Ang II) infusion‐induced sporadic TAA/D in Apoe^‐/‐^ mice.^9^ Although these previous studies revealed that the elevation of ADAMTS‐4 may contribute to arterial aneurysm formation, its role in AAA progression is not clear. Interestingly, we prior established an Ang II‐induced murine AAA model as described before,^10^, ^11^ and analysed the gene expression profile in the aneurysmal tissues and control aortas. The microarray profiling data showed a fourfold increase in ADAMTS‐4 in the aortic tissues of AAA mice compared to control aortas (*P* < .01). This finding inspired us to further elucidate ADAMTS‐4’s role in AAA formation.

MicroRNA‐126a‐5p (miR‐126a‐5p) is a 21‐nt microRNA that has a high sequence conservation between different species. Like other miRNAs, it acts a negative regulator for genes by degrading mRNA and/or blocking the translation process.^12^ Studies on miR‐126a‐5p in AAA formation are scarce; however, several literatures have revealed its involvement in artery diseases. Schober et al found that miR‐126a‐5p prevented endothelial cell function from hyperlipidaemia in an experimental atherosclerosis animal model.^13^ Similar protective effects of miR‐126a‐5p on vascular endothelium have also be demonstrated by Esser et al.^14^ In addition to analysing the mRNA profiles, our group also obtained the miRNA expression profiles in mouse aortic tissues. We found that miR‐126a‐5p decreased by 18‐fold in AAA tissues. Interestingly, we noted potential binding sites on the 3’ untranslated region (UTR) of both *mus* and *homo* ADAMTS‐4 for miR‐126a‐5p. Furthermore, miR‐126a‐5p expression was negatively correlated to ADAMTS‐4 in the analysed aortic samples. These findings promoted us to study the role of the dysregulated miR‐126a‐5p‐ADAMTS‐4 axis in AAA formation.

In this study, experimental AAA formation was induced by Ang II infusion in Apoe^‐/‐^ mice. To explore how the down‐regulated miR‐126a‐5p affects AAA development, its exclusive agomirs were given to Ang II‐infused mice. Our work shows that re‐expression of miR‐126a‐5p promotes the survival of mice injected with Ang II. On Ang II infusion, ADAMTS‐4 expression increases, which is inhibited by miR‐126a‐5p. Dual‐luciferase reporter results confirm that miR‐126a‐5p directly targets ADAMTS‐4.

## 2 MATERIALS AND METHODS

### 2.1 Ethic statement

We performed the animal experiments according to the Guide for the Care and Use of Laboratory Animals (National Institutes of Health). The Ethic Committee of Dalian Medical University has approved our study.

### 2.2 Ang II infusion AAA model and miRNA agomir administration

AAA was induced in Apoe^‐/‐^ mice according to the methods reported by Daugherty et al.^10^ In short, male Apoe^‐/‐^ mice (15 weeks; C57BL/6J background) were infused with 1 μg/kg/min Ang II (GL BioChem) or normal saline (Dubang Pharmaceutical Co., Ltd) with Model 2004 Alzet Osmotic minipumps (Alzet) that were implanted subcutaneously. Mice were anaesthetized via 2%‐3% isoflurane before surgical procedure. The formation of arterial aneurysm was defined by a 50% or greater dilation in the external diameter of suprarenal aorta.

Either miR‐126a‐5p (MIMAT0000137: 5’CAUUAUUACUUUUGGUACGCG3’) or NC agomirs (both 20 mg/kg) were intravenously injected into mice infused with Ang II. Four injections were performed at 0, 7, 14 and 21 days post the implantation of minipumps. MiRNA agomirs were obtained from GenePharma. (Shanghai).

For survival test, a total of 36 mice were included (n = 6 in sham group, n = 12 in AAA + miR‐126a‐5p agomirs, n = 18 in AAA + NC agomirs). The mortality was recorded for 28 d. Aortic tissues from survived mice were used in analysis of morphological changes. Additional 12 mice (n = 4 per group) were subjected to analyse protein and mRNA alterations of targeted genes.

### 2.3 Dual‐luciferase reporter assay

Measurement of normalized firefly luciferase activity was performed by using the pmirGLO Dual‐Luciferase miRNA Target Expression Vector (Promega Corporation) as per manufacturer's recommendations. Dual‐luciferase reporter constructs containing the 3’UTR of ADAMTS‐4 with miR‐126a‐5p binding sites were cotransfected with NC or miR‐126a‐5p agomirs into cells. Corresponding mutant 3’UTR fragments were also inserted into pmirGLO plasmid. Forty‐eight hours post the transfection, cells were analysed for luciferase. For each transfection, the firefly luciferase activity was normalized to renilla luciferase activity, and the luciferase activity was averaged from three replicates.

### 2.4 Aortic diameter measurement

Mouse aortic diameters were measured via GE volusonE8 ultrasound system at baseline and day 28 post‐aneurysm induction.

### 2.5 Human aortic smooth muscle cells (hASMCs) and treatment

The hASMCs were obtained from Z.q.x.z Cell Research and kept in ScienCell smooth muscle culture medium. For some experiment, hASMCs were stimulated with different doses of Ang II (0.5, 1 or 5 μM) for 12 hours or with 1 μM Ang II for 6, 12 or 24 hours.

### 2.6 Lentivirus (LV) vector‐mediated miRNA and gene expression in hASMCs

Precursor mir‐126 (pre‐mir‐126) and anti‐miR‐126‐5p sponge were inserted into lentivirus vectors, and packaged in 293T cells. The hASMCs were infected with LV‐pre‐mir‐126 (MOI = 10) or LV‐anti‐miR‐126‐5p sponge (MOI = 10) for 72 hours, and then stimulated with 1 μM Ang II for 24 hours.

### 2.7 RNA quantification via Real‐time quantitative PCR (real‐time qPCR)

Total RNAs were isolated and reversely transcribed into cDNAs via a specific adaptor primers for miR‐126a‐5p (5’ GTTGGCTCTGGTGCAGGGTCCGAGGTATTCGCACCAGAGCCAACCGCGTA3’) and the control 5s (*homo*: 5’ GTTGGCTCTGGTGCAGGGTCCGAGGTATTCGCACCAGAGCCAACAAAGCCTAC3’; *mus*: GTTGGCTCTGGTGCAGGGTCCGAGGTATTCGCACCAGAGCCAACTAGCCTTGC3’) for detection the expression of miR‐126a‐5p. For other genes, RNAs were processed into cDNA in presence of oligo (dT)_15_ and random primers. We carried the reverse transcription experiments by using Taq PCR MasterMix (BioTeke Bio). RNA expression was quantitatively analysed using SYBR Green miRNA assays (Sigma‐Aldrich) as per the manufacturer's protocols. Primers used in real‐time qPCR were listed in table 1. The relative expression levels were presented as 2^−ΔΔ^ *^C^* ^t^.

**Table 1:** Primers

Names	Sequences
MiR‐126a‐5p	Forward: 5’ CATTATTACTTTTGGTACGCG 3’
	Reverse: 5’ GCAGGGTCCGAGGTATTC 3’
*homo* 5s	Forward: 5’GATCTCGGAAGCTAAGCAGG 3’
	Reverse: 5’ GCAGGGTCCGAGGTATTC 3’
*mus* 5s	Forward: 5’ CTAAAGATTTCCGTGGAGAG 3’
	Reverse: 5’ GCAGGGTCCGAGGTATTC 3’
*homo* ADAMTS‐4	Forward: 5’ CCCAAGCATCCGCAATCC3’
	Reverse: 5’CAGGTCCTGACGGGTAAACA3’
*mus* ADAMTS‐4	Forward:5’TGGCAAGTATTGTGAGGGC3’
	Reverse: 5’GGTTCGGTGGTTGTAGGC3’
*mus* MMP‐9	Forward:5’GCAACGGAGAAGGCAAAC3’
	Reverse: 5’CACTCGGGTAGGGCAGAA3’
*mus* MMP‐2	Forward:5’CCCCGATGCTGATACTGAC3’
	Reverse: 5’CTGTCCGCCAAATAAACC3’
*mus* MMP‐13	Forward:5’GATGAAACCTGGACAAGC3’
	Reverse: 5’CTGGACCATAAAGAAACTGA3’
*mus* MMP‐16	Forward:5’GGACCAACAGACCGAGAT3’
	Reverse: 5’CCAATACAAGGAGGCATAA3’
*homo* β‐actin	Forward:5’GGCACCCAGCACAATGAA3’
	Reverse: 5’TAGAAGCATTTGCGGTGG3’
*mus* β‐actin	Forward:5’CTGTGCCCATCTACGAGGGCTAT3’
	Reverse: 5’TTTGATGTCACGCACGATTTCC3’

### 2.8 Western blot

Protein samples were collected from aortic tissues and hASMCs by using Cell lysis buffer containing 1 mM phenylmethylsulfonyl fluoride (Beyotime). After being separated on SDS‐PAGE, proteins were transferred onto polyvinylidene fluoride (PVDF) membranes (Millipore) and incubated with 5% (M/V) skim milk for 60 min. Primary antibodies including anti‐ADAMTS‐4 polyclonal antibody (PcAb) (ABclonal, Wuhan, China; a dilution of 1:1000), anti‐collagen‐I monoclonal antibody (McAb) (Abcam, Cambridge, MA, USA; a dilution of 1:1000) and anti‐Elastin antibody PcAb (ABclonal, a dilution of 1:500) were used to probe corresponding antigens. The PVDF membranes were incubated with one of the above antibodies at 4°C overnight. Thereafter, horseradish peroxidase (HRP)‐conjugated secondary antibodies (Beyotime, a dilution of 1:5000) was used to incubate the membranes. Protein blots were visualized with BeyoECL Plus (Beyotime), and their intensities were detected via Gel Pro‐analyzer software. The levels of target proteins were normalized to β‐actin.

### 2.9 Morphological changes

Mouse aortas were collected at day 28 post‐Ang II or saline fusion, fixed in 4% paraformaldehyde, and embedded into paraffin, cut into 5‐μm sections and subjected to H&E staining (Solarbio; Sangon). Antigens were retrieved from aortic slices by citric acid. After washing with 1 × PBS for three times, tissue sections were blocked with goat serum for 15 min at room temperature. For immunofluorescent (IF) assay, tissue sections were incubated with anti‐ADAMTS‐4 PcAb (Abcam; a dilution of 1:200), anti‐CD68 (Santa Cruz Biotechnology, Inc; a dilution of 1:50) and anti‐SM‐22α (Abcam; a dilution of 1:100) at 4°C overnight. Cy3‐marked IgG (Beyotime; dilution of 1:200) was then used to incubate the tissue slices for 1.5 hours. Fluorescent pictures were then taken under a fluorescent microscope. To stain elastic laminas, aortic tissue slices were stained with Elastin van Gieson (EVG) reagent for 15 minutes.

### 2.10 Enzyme‐Linked Immunosorbent Assay (ELISA)

Protein levels of interleukin 6 (IL‐6) and monocyte chemoattractant protein‐1 (MCP‐1) were determined with ELISA kits (LIANKE Biotech).

### 2.11 Statistics

GraphPad Prism software version 8.0 (GraphPad Software) was used to analyse all data. Values were presented as means ± SD or SEM. Kaplan‐Meier curve followed by log‐rank (Mantel‐Cox) test was used to analyse the survival data. Data from two groups were analysed with Student's *t* test, while data from multiple groups were analysed with one‐way ANOVA followed by Tukey's multiple comparisons test. Cell counts in IF staining of aortic tissues were analysed with Kruskal‐Wallis test followed by Dunn's multiple comparison test. *P* < .05 was considered statistically significant.

## 3 RESULTS

### 3.1 Ang II infusion alters the expression miR‐126‐5p and ADAMTS‐4 in mouse aorta tissues

**Figure 1:**
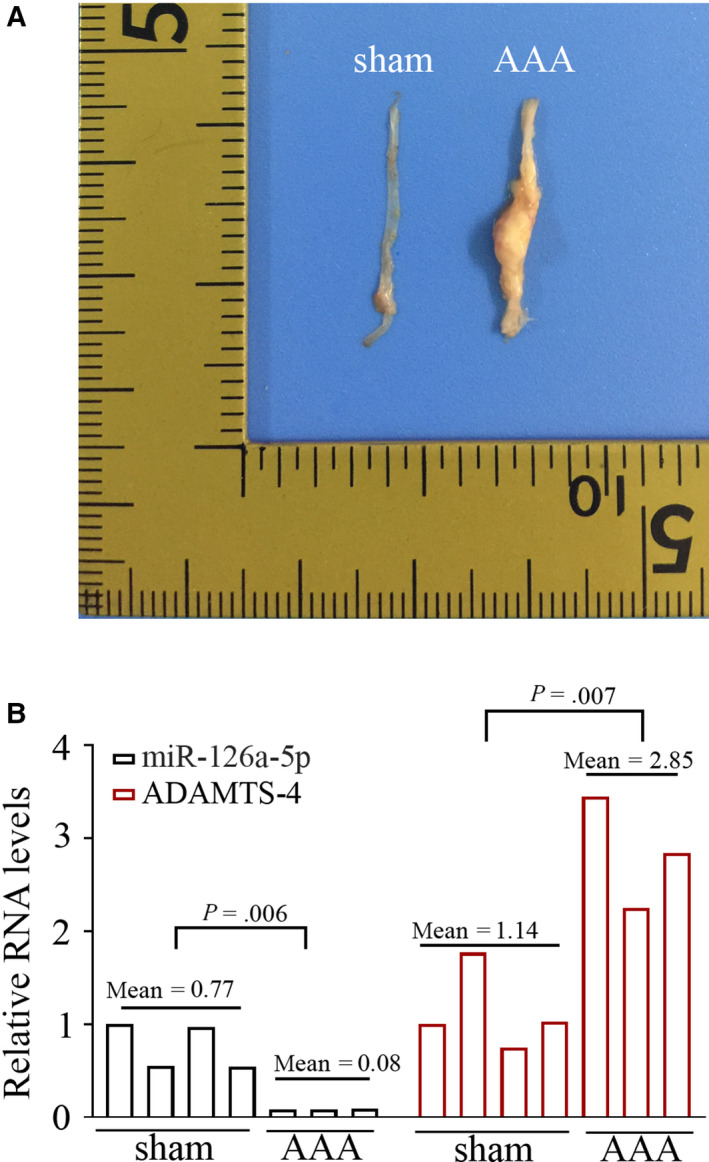
Ang II infusion alters the expression miR‐126a‐5p and ADAMTS‐4 in mouse aorta tissues Ang II was infused into Apoe^‐/‐^ mice for continuous 28 d via osmotic minipumps. On day 28, the survival mice were killed, and representative aortas were shown in (A). The expression of miR‐126a‐5p and ADAMTS‐4 was determined with real‐time qPCR (B). *P* < .05 was considered statistically significant

Ang II was infused into Apoe^‐/‐^ mice for continuous 28 d via osmotic minipumps. On day 28, the survival mice were killed, and type III‐IV arterial aneurysms (Figure 1A) were collected for microarray analysis (n = 3). Aorta tissues from four sham‐operated mice (Figure 1A) were collected as the control for microarray assay (n = 4). Real‐time qPCR data illustrated that miR‐126a‐5p expression was significantly lower in AAA tissues than in control aortas (Figure 1B). ADAMTS‐4 showed opposite expression pattern to miR‐126a‐5p (Figure 1B). These data demonstrate that Ang II infusion alters the expression of miR‐126a‐5p and ADAMTS‐4 to opposite directions in mouse abdominal aortas. In addition, genes potentially targeted by and negatively expressed (Pearson correlation coefficient <−0.7, and *P* value <.05) with miR‐126a‐5p in mouse aortic tissues (n = 4 for sham, n = 3 for AAA) were listed in Table S1.

### 3.2 Delivery of miR‐126a‐5p limits AAA formation

**Figure 2:**
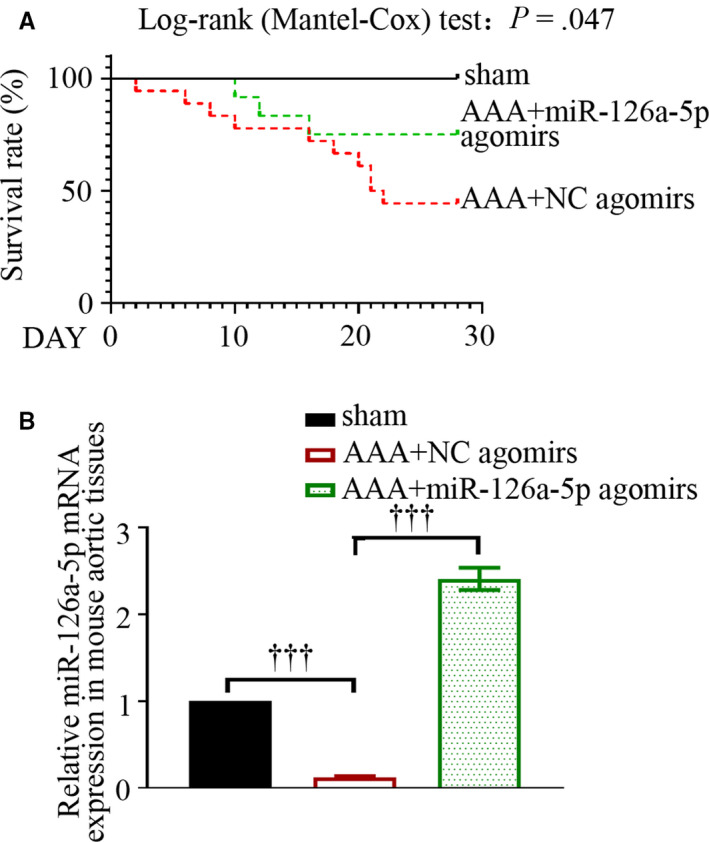
MiR‐126a‐5p agomirs improve the survival of mice treated with Ang II. Kaplan‐Meier curve followed by log‐rank (Mantel‐Cox) test was performed to assess the survival data (A). *P* < .05 was considered statistically significant. The expression levels of miR‐126a‐5p in mouse aortas were determined with real‐time qPCR on day 28 (B). Values were presented as means ± SEM (n = 4). ^†††^ *P* < .001

**Figure 3:**
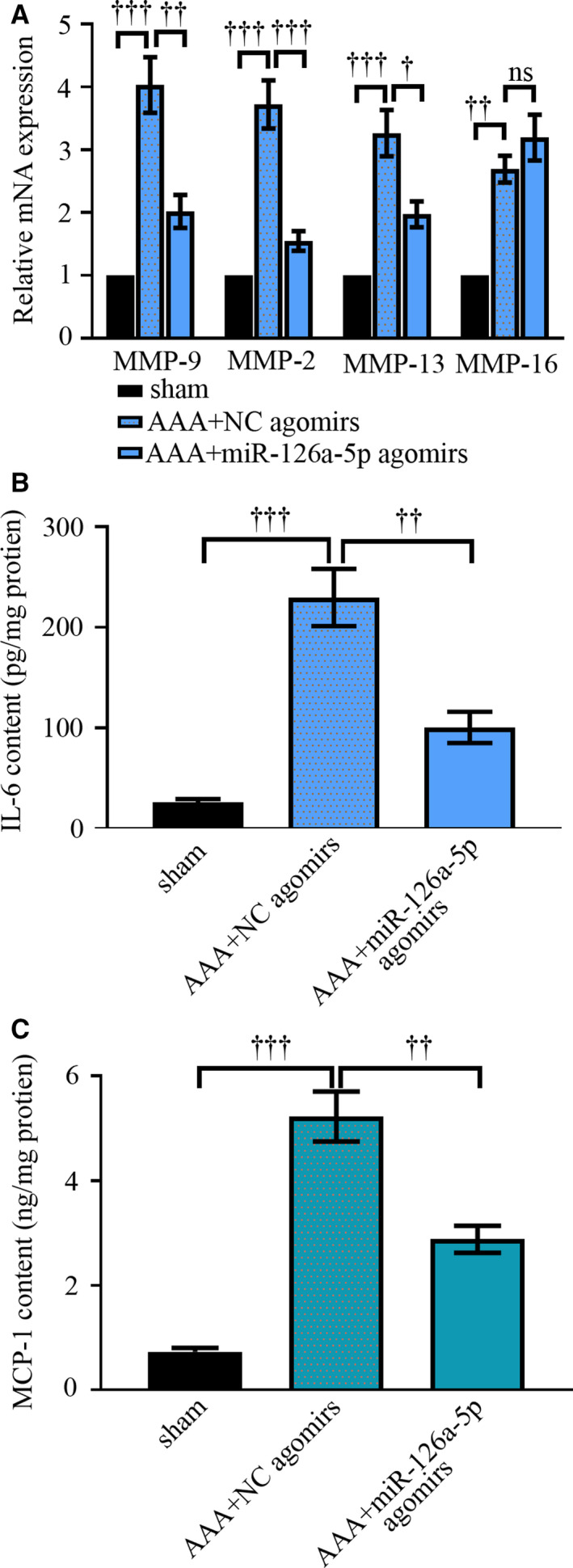
MiR‐126a‐5p agomirs alter cytokine expression in aortic tissues. Ang II was infused into Apoe^‐/‐^ mice for continuous 28 d via osmotic minipumps. On day 28, mice were killed. mRNA expression levels of MMP‐9, MMP‐2, MMP‐13 and MMP‐16 were determined with real‐time qPCR (A). Protein levels of IL‐6 and MCP‐1 were determined with ELISA (B‐C). Values were presented as means ± SEM (n = 4). ^†††^ *P* < .001, ^††^ *P* < .01, ^†^ *P* < .05, ^ns^ *P* > 0.05

To mediate the overexpression of miR‐126a‐5p in mouse aortas, four intravenous injections of 20 mg/kg miR‐126a‐5p agomirs were given to mice. We noted that miR‐126a‐5p agomirs, but not NC agomirs, significantly improved the survival of mice infused with Ang II (Figure 2A; *P* < .05). The delivery of miR‐126a‐5p agomirs could restore miR‐126a‐5p expression in Ang II‐infused aortas (Figure 2B). Data from real‐time qPCR showed that miR‐126a‐5p overexpression reduced MMP‐9, MMP‐2 and MMP‐13 expression in Ang II‐infused aortas (Figure 3A). Further, the generation of pro‐inflammatory cytokines IL‐6 and MCP‐1 decreased following miR‐126a‐5 delivery in vivo (Figure 3B‐C).

**Figure 4:**
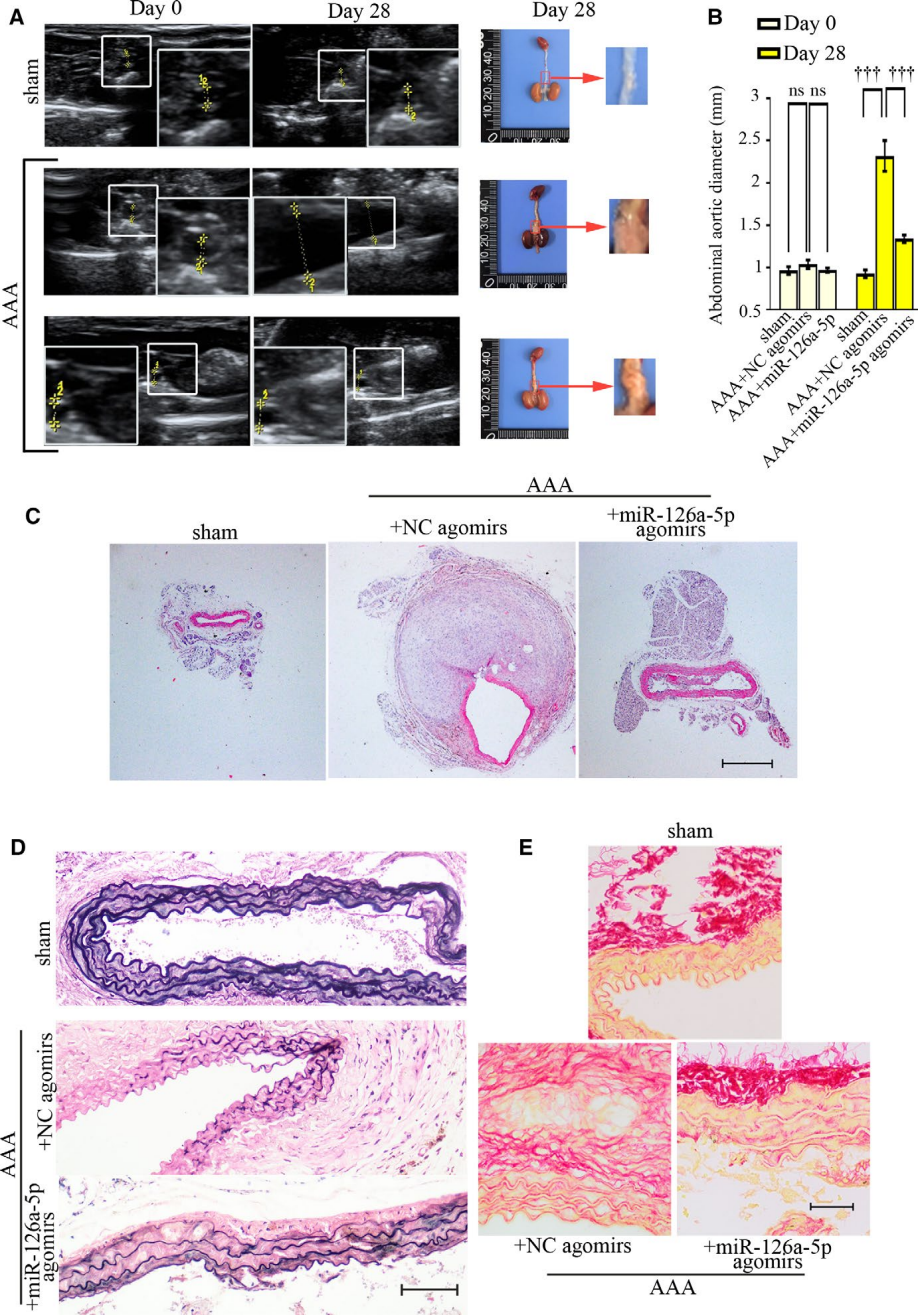
MiR‐126a‐5p agomirs limits AAA formation in vivo. AAA mice were treated with miR‐126a‐5p agomirs or NC agomirs, and on day 28 post‐Ang II treatment, all mice were killed. Representative ultrasound images (longitudinal view) showing suprarenal aortas of Apoe^‐/‐^ mice, and the maximal aortic diameters were recorded (A, left; B). Whole mouse aortas were shown in A (right). Values were presented as means ± SEM (n = 5). ^†††^ *P* < .001. Aortas were stained via H&E (C; Bar, 500 μm), and EVG (D; Bar, 100 μm). The deposition of collagens was probed with Sirius red (E; Bar, 50 μm). ^†††^ *P* < .001

Representative longitudinal ultrasound images (Figure 4A, left) and the picture of whole mouse aortas (Figure 4A, right) revealed that Ang II‐induced increase in aorta diameter was attenuated by miR‐126a‐5p agomirs (Figure 4B). Abdominal aortas were then stained with H&E (Figure 4C), and EVG (Figure 4D). The staining images clearly indicated a degradation of elastic laminas in Ang II‐infused aortas. As compared to AAA mice injected with NC agomirs, mice administrated with miR‐126a‐5p agomirs had increased layers of elastic laminas. The integrity of elastic laminas was also impaired by Ang II, which was protected by miR‐126a‐5p agomirs as well. Sirius red could stain collagens in yellow and red. As indicated in Figure 4E, miR‐126a‐5p agomirs attenuated the degradation of collagens induced by Ang II injection. These data together reveal a protective role of miR‐126a‐5p in experimental AAA formation.

### 3.3 ADAMTS‐4 increases in response to Ang II infusion, whereas decreases when miR‐126a‐5p is overexpressed

**Figure 5:**
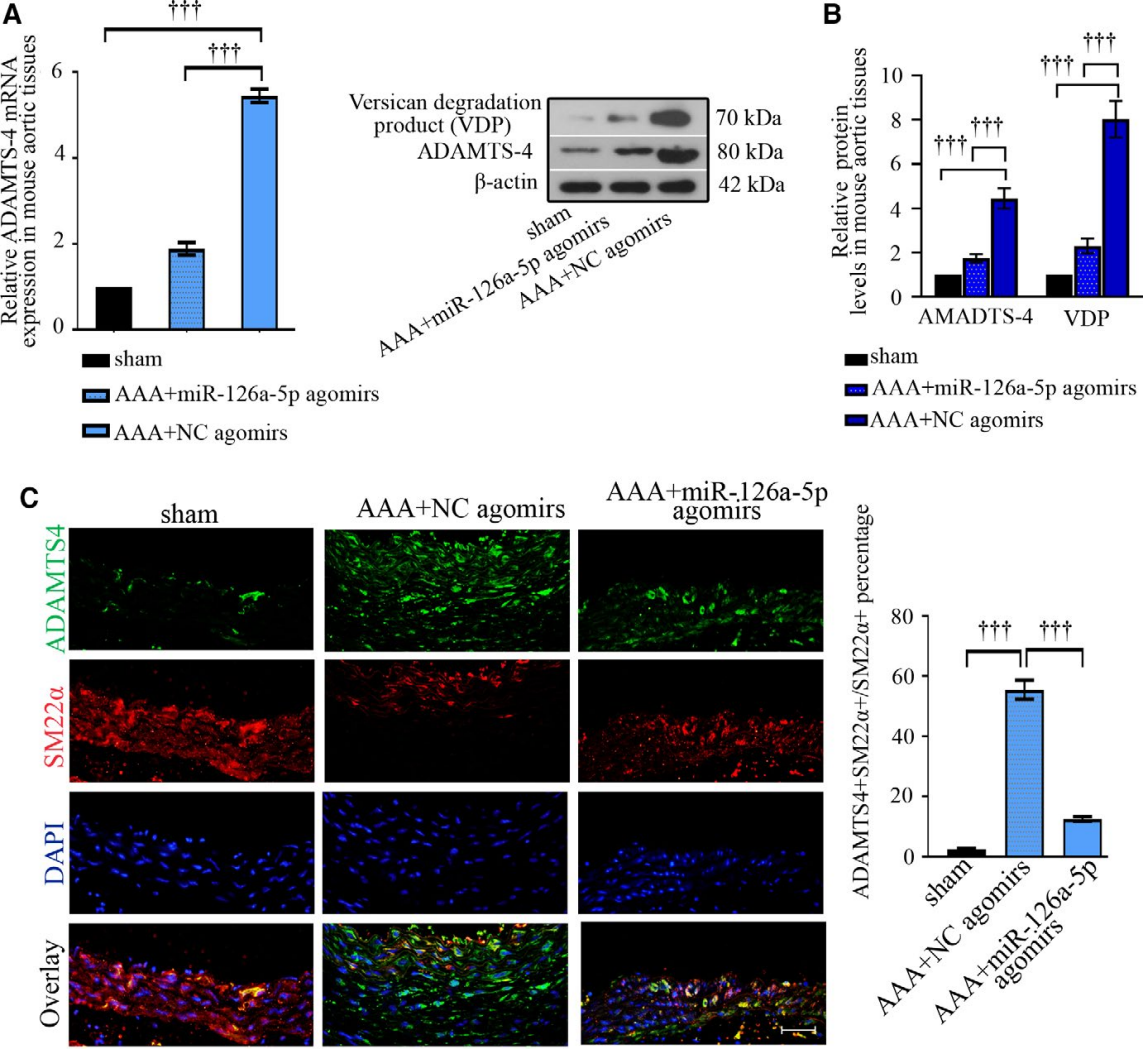
ADAMTS‐4 expression up‐regulates in aortic SMCs upon Ang II infusion, whereas is decreased by miR‐126a‐5p agomirs in vivo. The mRNA and protein expression of ADAMTS‐4 or versican degradation products (VDP) were analysed with real‐time qPCR (A) and Western blot (B). Values were presented as means ± SEM (n = 4). The protein expression levels of ADAMTS‐4 (green) and SM‐22α (red, a marker for vascular SMCs) were also probed with immunofluorescent assay and quantified (C). Bar, 50 μm. Values were presented as means ± SEM (n = 6 for sham, n = 8 for AAA + NC agomirs, n = 9 for AAA + miR‐126a‐5p agomirs).^†††^ *P* < .001

ADAMTS‐4 mRNA and protein levels were up‐regulated by Ang II infusion and down‐regulated by miR‐126a‐5p overexpression in mouse aortic tissues (Figure 5A‐B). To determine what type of cells expresses ADAMT‐4, SMCs were marked via SM‐22α and macrophages were labelled with CD68. Immunofluorescent images showed that, in response to Ang II infusion, a strong up‐regulation of ADAMTS‐4 presented in SMCs of aortic media (Figure 5C). An elevation of ADAMT‐4 was also observed in macrophages infiltrated in the arterial aneurysm (Figure S1). Immunofluorescent data validate the down‐regulation in ADAMTS‐4 can be induced by miR‐126a‐5p agomirs.

### 3.4 Leniviruses expressing pre‐mir‐126 reduces Ang II‐induced elevation in ADAMTS‐4 in primary hASMCs in vitro

**Figure 6:**
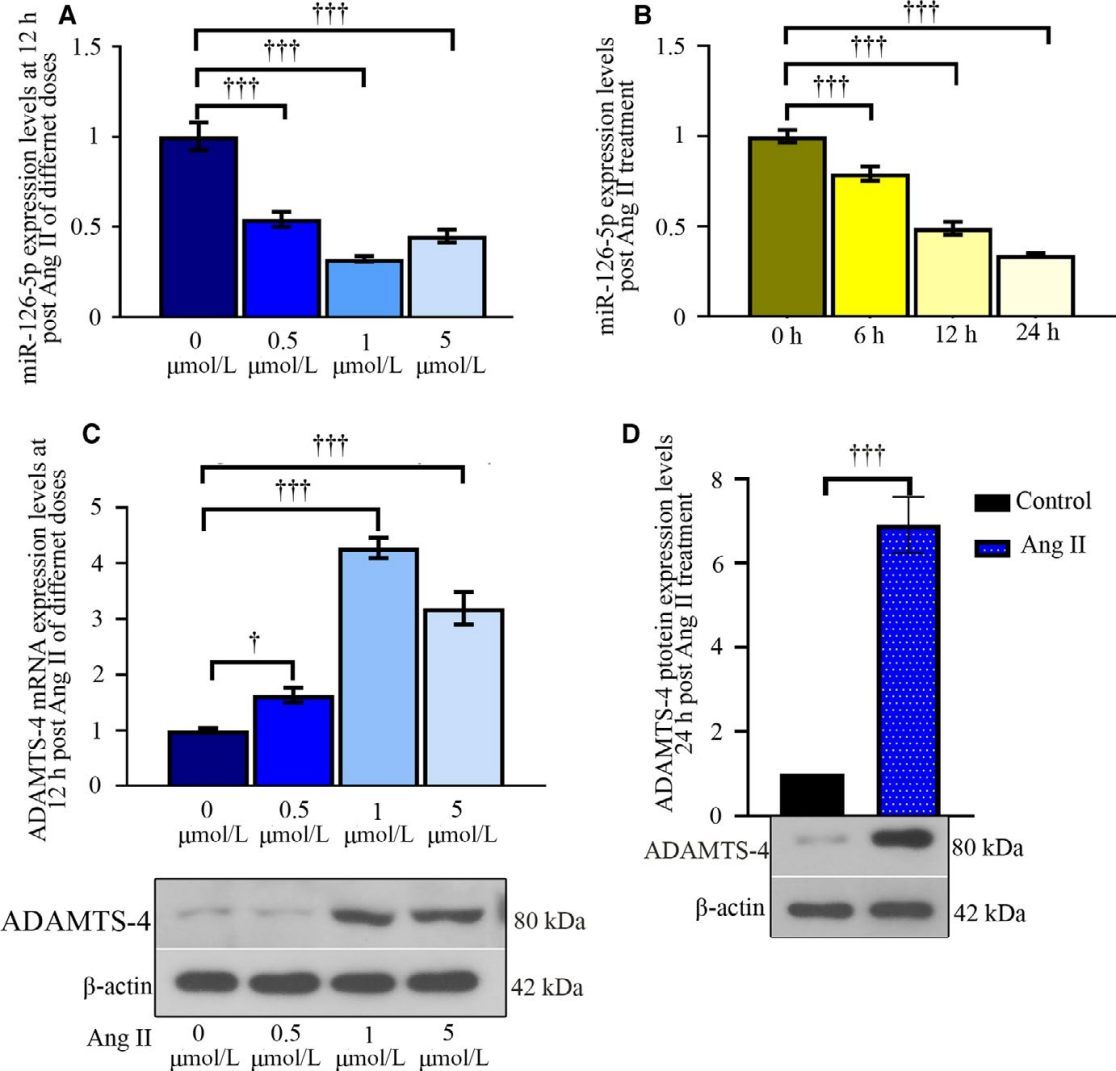
Ang II alters the expression of miR‐126‐5p and ADAMTS‐4 towards opposite directions in hASMCs. Primary hASMCs were exposed to indicated doses of Ang II for 12 h, and the levels of miR‐126‐5p and ADAMTS‐4 were determined with real‐time qPCR (A & B). Then, cells were treated with 1 μM Ang II for indicated time periods, and the expression of miR‐126‐5p was determined (C). The protein expression of ADAMTS‐4 was determined at 24‐h post 1 μM Ang II exposure (D). Values were presented as means ± SD (n = 3). ^†††^ *P* < .001

**Figure 7:**
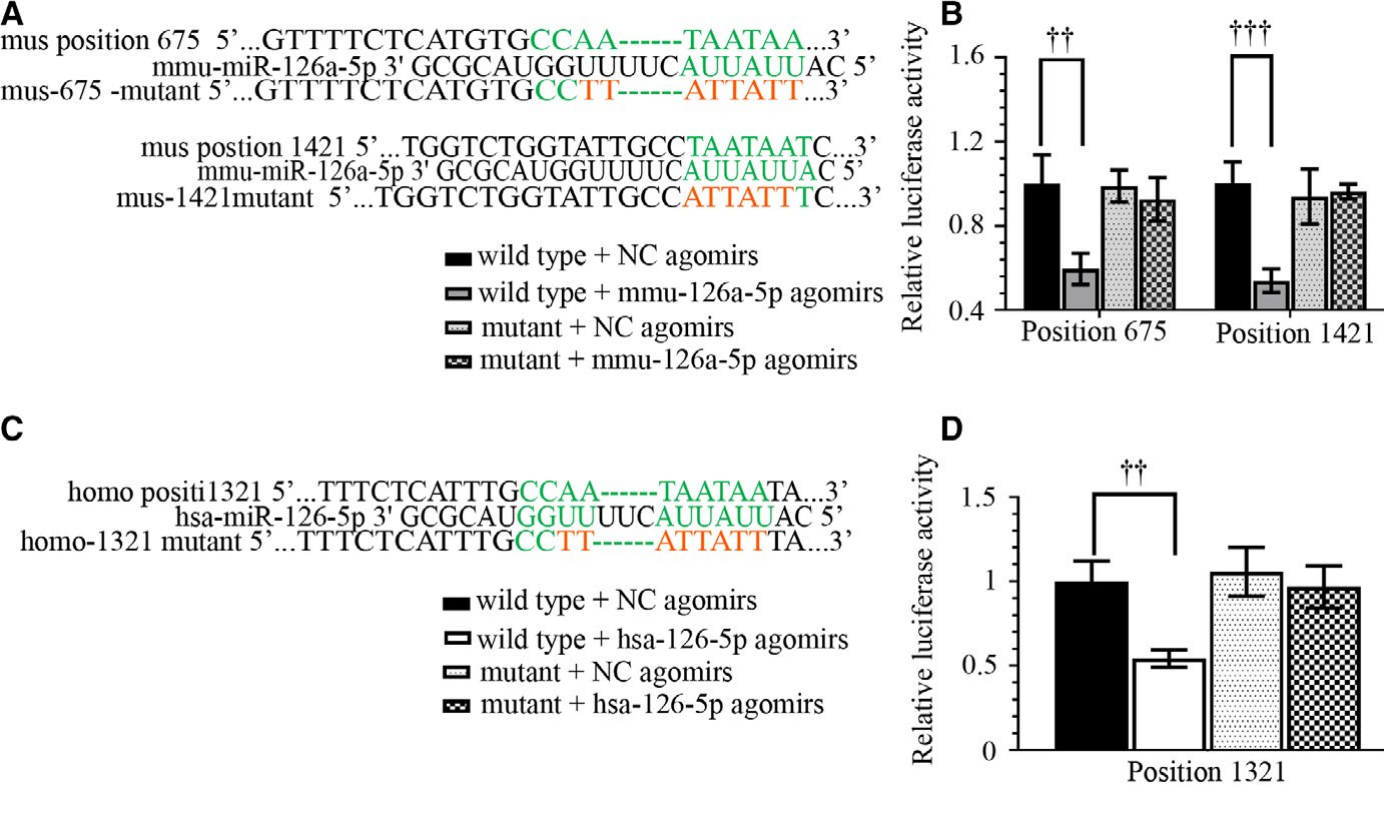
ADAMTS‐4 is a direct target for miR‐126‐5p. The sequence information of wild‐type and mutant type ADAMTS‐4 3’UTR was listed in (A & C). Dual‐luciferase reporter assay was performed to analyse the binding between miR‐126‐5p and ADAMTS‐4 (B & D). Values were presented as means ± SD (n = 3). ^††^ *P* < .01, ^†††^ *P* < .001

**Figure 8:**
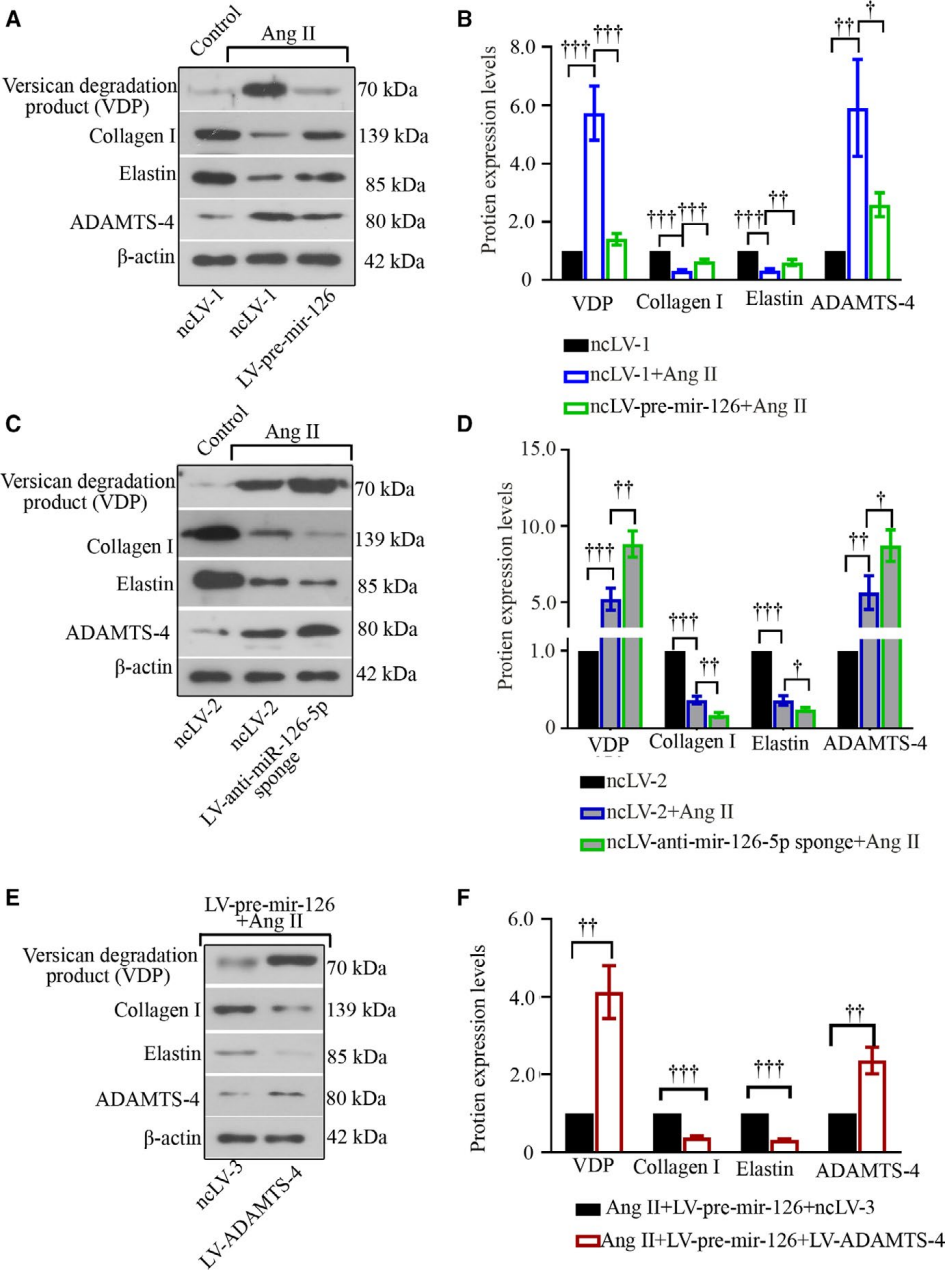
MiR‐126‐5p inhibits Ang II‐induced ECM loss and reduces ADAMTS‐4 in hASMCs. Primary hASMCs were infected with pre‐mir‐126 or anti‐miR‐126‐5p sponge lentiviruses before Ang II stimulation (A‐D). Re‐expression of ADAMTS‐4 was also mediated with lentiviruses (E‐F). The expression levels of versican degradation products, collagen I, elastin and ADAMTS‐4 were determined with Western blot (A‐F). Values were presented as means ± SD (n = 3). ^†^ *P* < .05, ^††^ *P* < .01, ^†††^ *P* < .001

In keeping with in vivo data, Ang II decreased miR‐126‐5p expression in a dose‐ and time‐dependent way in hASMCs (Figure 6A‐B). Ang II altered the expression of miR‐126‐5p and ADAMTS‐4 towards opposite directions—ADAMTS‐4 expression and versican degradation products markedly increased (Figure 6C‐D). There were two potential binding sites on *mus* ADAMTS‐4 3’UTR and one site on *homo* ADAMTS‐4 3’UTR for mmu‐miR‐126a‐5p/hsa‐miR‐126‐5p (Figure 7A & C). Additional relative luciferase data demonstrated ADAMTS‐4 as a novel target for miR‐126a‐5p (Figure 7 B & D). Moreover, Ang II‐induced ADAMTS‐4 up‐regulation was suppressed by pre‐mir‐126 viral infection, but augmented by anti‐miR‐126‐5p sponge viral infection (Figure 8A‐D). MiR‐126‐5p increased collagen I generation and elastin expression, and reduced versican degradation in hASMCs treated with Ang II (Figure 8A‐D). Re‐expression of ADAMTS‐4 accelerated Ang II‐induced alterations in cells overexpressing pre‐mir‐126 (Figure 8E‐F). Such data demonstrate that miR‐126‐5p prevents ECM degradation induced by Ang II in hASMCs through targeting ADAMTS‐4.

## 4 DISCUSSION

In the present study, miR‐126a‐5p or NC agomirs were given to mice infused with Ang II intravenously. As compared to sham‐operated animals, mice infused with Ang II displayed classic features of AAA, including obvious formation of aneurysm, aortic dilatation, fragment and degradation of elastic laminas. These pathological alterations were attenuated by miR‐126a‐5p overexpression. ADAMTS‐4 down‐regulation contributed to the anti‐AAA effects of miR‐126a‐5p.

By performing fluorescence in situ hybridization (FISH) assay, we found that this miRNA was detectable in the normal aortic wall and mainly expressed in tunica media (data not shown). After AAA induction, its expression decreased in aortic wall. The sequence of mmu‐miR‐126a‐5p and has‐miR‐126‐5p is identical (5’CAUUAUUACUUUUGGUACGCG3’). The high conservation of miR‐126(a)‐5p between human and model animals endows our work performed in mice a clinical significance. We observed a down‐regulation of miR‐126(a)‐5p in Ang II‐treated mouse aortas and hASMCs. Both mature miR‐126a‐5p (previous name: miR‐126*) and miR‐126a‐3p (miR‐126) are generated from precursor mir‐126a (pre‐mir‐126a) via dicer.^15^ Hua et al found that Ang II reduced miR‐126‐3p in vascular SMCs in vitro.^16^ Such findings together with ours suggest that Ang II may interfere the processing of primary‐mir‐126 (pri‐mir‐126) or pre‐mir‐126, thereby promoting down‐regulation in both mature miRNAs. The sequences of miR‐126‐5p and miR‐126‐3p are distinctly different with each other, which makes their target gene profiles quite different. While the anti‐AAA role of miR‐126‐3p has been reported before,^17^ miR‐126‐5p's role in AAA formation is unknown. Inspired by our prior microarray data, we treated mice with miR‐126a‐5p agomirs to up‐regulate miR‐126a‐5p expression in Ang II‐infused aortas. Our work for the first time reveals that miR‐126a‐5p improves the survival of mice treated with Ang II, reduces the degradation of elastic laminas and arrests AAA formation.

Matrix metalloproteinases (MMPs) are a family of enzymes contributing to ECM degeneration and remodelling in AAA development.^18^ Unlike the well‐defined pathogenic role of MMPs reported in the development of AAA,^18^, ^19^ the role of some ADAMTSs is conflicted in aneurysmal diseases. Vorkapic et al found that several members of the ADAMTS family, including ADAMTS‐1, ADAMTS‐4, ADAMTS‐5, ADAMTS‐8, ADAMTS‐9, were down‐regulated in human AAA tissues compared to control aortas.^20^ In contrast, other investigators observed increases in these ADAMTSs in aortic wall, serum or macrophage samples collected from patients with AAA.^21^, ^22^, ^23^ ADAMTS‐5 deficiency exacerbated aortic dilatation induced by Ang II,^24^ while ADAMTS‐4 deficiency attenuated Ang II‐induced TAA/D, inhibited proteoglycan degradation and elastin fragment.^9^ As our microarray results suggested that ADAMTS‐4 expression was negatively correlated to miR‐126a‐5p in aneurysms and control aortas in mice, we then investigated whether miR‐126a‐5p could down‐regulate ADAMTS‐4.

Ren and co‐workers found that ADAMTS‐4 overexpressed in the both aortic media and adventitia in mice challenged with Ang II.^9^ Our data in consistent with their study demonstrated an up‐regulation of ADAMTS‐4 aortic wall in AAA mice. Degeneration and disorganization of the elastic media is the key histopathological feature for AAA development.^25^, ^26^ Dysfunction of ASMCs contributes to the aortic dilatation and eventual rupture in progressed AAA.^27^ Elastic fibres fail to form normal elastic lamina‐SMC connections due to a local elevation of Ang II signalling.^28^ Labelling cells with SM‐22α antibody enabled us to observe an elevation of ADAMTS‐4 in aortic SMCs in mice. By staining the elastic fibres with EVG, we observed that up‐regulation of miR‐126a‐5p prevented elastin from degradation. We next treated hASMCs with Ang II at different doses. Ang II treatment directly reduced the expression of miR‐126‐5p and increased ADAMTS‐4 in cultured hASMCs. These data in agreement with our in vivo results suggested these two molecules as Ang II sensitive, and were negatively correlated. We demonstrated both *mus* and *homo* ADAMTS‐4 as the direct and novel target for miR‐126(a)‐5p via dual‐luciferase reporter assay. Additional gain‐ and loss‐of‐function studies performed in hASMCs exposed to Ang II further proved that miR‐126‐5p acted as a negative regulator for ADAMTS‐4. In vitro, ADAMTS‐4 overexpression attenuated the anti‐ECM degradation effects of miR‐126a‐5p in aortic SMCs exposed to Ang II. Hence, it is plausible that ADAMTS‐4 expression may overcome the miR‐126‐5p effect in vivo. Lack of in vivo delivery of ADAMTS‐4 was one limitation of the present study and should be performed in the future.

Of note, Ren et al^9^ found a colocation of ADAMTS‐4 in macrophages infiltrated in aortas, which was supported by our findings. Moreover, they demonstrated the expression of ADAMTS‐4 contributed to enhanced infiltration of pro‐inflammatory macrophages during arterial aneurysm formation. The correlation of ADAMTS‐4 up‐regulation with inflammation has been reported in other human diseases associated with inflammation.^29^, ^30^ However, a previous study also reported an anti‐inflammatory role of ADAMTS‐4 in the central nervous system.^31^ Although we performed the present work to investigate the role of miR‐126a‐5p‐ADAMTS‐4 axis in regulation ECM homeostasis, to understand its role in AAA‐associated inflammation is needed.

MMP‐9 and MMP‐2 are the two most widely studied MMPs because of their ability to degrade elastin and collagen.^19^, ^32^ Interestingly, we noted that the expression of these two MMPs decreased following administration of miR‐126‐5p agomirs. However, several online databases (TargetScan, miRDB or Starbase) predict MMP‐13 and MMP‐16, but not MMP‐9 and MMP‐2, as potential targets for miR‐126a‐5p. These findings suggest that miR‐126‐5p may not regulate their expression directly. Reduction of MMP‐9 and MMP‐2 may occur when AAA was attenuated by miR‐126‐5p. We also determined the expression of MMP‐13 and MMP‐16, and observed that only MMP‐13 expression decreased following miR‐126a‐5p overexpression. Such data suggest that, aside from ADAMTS‐4, miR‐126a‐5p may also attenuate ECM degradation by directly targeting MMPs. In addition, Ventricular Zone Expressed PH Domain Containing 1 (VEPH1) was identified as a novel regulator of phenotypic switch of vascular SMCs by our group (data not shown), and it was a predicted target of miR‐126a‐5p. To further investigate whether miR‐126a‐5p/VEPH1 axis plays a role in phenotypic transition of aortic SMCs will help to fully evaluate the anti‐AAA effects of miR‐126a‐5p.

We understand that ADAMTS‐1, ADAMTS‐4 and ADAMTS‐5 are the major proteases responsible for the cleavage and degradation of hyaluronan‐binding proteoglycans,^6^ but not collagen I or elastin. However, a previous study from Hong‐Brown et al showed that silencing of ADAMTS‐1 blocked alcohol‐induced degradation of elastin in myocytes.^33^ Considering the important function of collagen I and elastin in maintaining the integrity of aortic wall, we further analysed their expression in Ang II‐treated hASMCs. Our data illustrated that miR‐126‐5p protected collagen I and elastin from Ang II‐induced degradation, and interestingly, ADAMTS‐4 inhibition was involved such protective effects. It has been suggested that ADAMTS‐4 is not able to directly cleave collagen I or elastin.^34^ We then asked why these two ECM components were reduced post‐ADAMTS‐4 overexpression. ADAMTS‐4 neutralizing antibody has been demonstrated to block pro‐inflammatory interleukin 1 (IL‐1) signalling transduction in chondrocytes,^35^, ^36^ suggesting a potential pro‐inflammatory role of ADAMTS‐4. Overgeneration of pro‐inflammatory cytokines, such as IL‐1, is also one major cause for ECM degradation in aneurysm formation.^37^ These findings together with our work imply that ADAMTS‐4 may indirectly contribute to collagen I and elastin degradation by inducing inflammation.

Collectively, our work demonstrates miR‐126a‐5p down‐regulation as a pathogenic factor in Ang II‐induced AAA formation. Overexpression of miR‐126a‐5p limits murine AAA formation, in which ADAMTS‐4 suppression is involved. This study suggests miR‐126a‐5p‐ADAMTS‐4 as a potential therapeutic target for AAA development.

## CONFLICT OF INTEREST

We have no conflict of interest.

## AUTHOR CONTRIBUTIONS

LL wrote the paper. LL, WM and SP performed the research. LL, YL, HW and BW analysed the data. LL and WM designed the research study. RK helped to design the research study and revised the manuscript. All authors approved the submission.

## Supporting information

Fig S1

Table S1

Supplementary Material

## Data Availability

All data are presented in this manuscript.

## References

[1] Weintraub NL . Understanding abdominal aortic aneurysm. N Engl J Med. 2009;361:1114‐1116. 19741234 10.1056/NEJMcibr0905244 PMC3791612

[2] Raaz U , Zollner AM , Schellinger IN , et al. Segmental aortic stiffening contributes to experimental abdominal aortic aneurysm development. Circulation. 2015;131:1783‐1795. 25904646 10.1161/CIRCULATIONAHA.114.012377 PMC4439288

[3] Kuivaniemi H , Ryer EJ , Elmore JR , Tromp G . Understanding the pathogenesis of abdominal aortic aneurysms. Expert Rev Cardiovasc Ther. 2015;13:975‐987. 26308600 10.1586/14779072.2015.1074861 PMC4829576

[4] Anidjar S , Salzmann JL , Gentric D , Lagneau P , Camilleri JP , Michel JB . Elastase‐induced experimental aneurysms in rats. Circulation. 1990;82:973‐981. 2144219 10.1161/01.cir.82.3.973

[5] Raffort J , Lareyre F , Clement M , Hassen‐Khodja R , Chinetti G , Mallat Z . Monocytes and macrophages in abdominal aortic aneurysm. Nat Rev Cardiol. 2017;14:457‐471. 28406184 10.1038/nrcardio.2017.52

[6] Rienks M , Barallobre‐Barreiro J , Mayr M . The emerging role of the ADAMTS family in vascular diseases. Circ Res. 2018;123:1279‐1281. 30566059 10.1161/CIRCRESAHA.118.313737

[7] Stanton H , Melrose J , Little CB , Fosang AJ . Proteoglycan degradation by the ADAMTS family of proteinases. Biochim Biophys Acta. 2011;1812:1616‐1629. 21914474 10.1016/j.bbadis.2011.08.009

[8] Ren P , Zhang L , Xu G , et al. ADAMTS‐1 and ADAMTS‐4 levels are elevated in thoracic aortic aneurysms and dissections. Ann Thorac Surg. 2013;95:570‐577. 23245439 10.1016/j.athoracsur.2012.10.084 PMC3593585

[9] Ren P , Hughes M , Krishnamoorthy S , et al. Critical role of ADAMTS‐4 in the development of sporadic aortic aneurysm and dissection in mice. Sci Rep. 2017;7:12351. 28955046 10.1038/s41598-017-12248-z PMC5617887

[10] Daugherty A , Manning MW , Cassis LA . Angiotensin II promotes atherosclerotic lesions and aneurysms in apolipoprotein E‐deficient mice. J Clin Invest. 2000;105:1605‐1612. 10841519 10.1172/JCI7818 PMC300846

[11] Saraff K , Babamusta F , Cassis LA , Daugherty A . Aortic dissection precedes formation of aneurysms and atherosclerosis in angiotensin II‐infused, apolipoprotein E‐deficient mice. Arterioscler Thromb Vasc Biol. 2003;23:1621‐1626. 12855482 10.1161/01.ATV.0000085631.76095.64

[12] Kumar S , Boon RA , Maegdefessel L , Dimmeler S , Jo H . Role of noncoding RNAs in the pathogenesis of abdominal aortic aneurysm. Circ Res. 2019;124:619‐630. 30763215 10.1161/CIRCRESAHA.118.312438 PMC6440479

[13] Schober A , Nazari‐Jahantigh M , Wei Y , et al. MicroRNA‐126‐5p promotes endothelial proliferation and limits atherosclerosis by suppressing Dlk1. Nat Med. 2014;20:368‐376. 24584117 10.1038/nm.3487 PMC4398028

[14] Esser JS , Saretzki E , Pankratz F , et al. Bone morphogenetic protein 4 regulates microRNAs miR‐494 and miR‐126‐5p in control of endothelial cell function in angiogenesis. Thromb Haemost. 2017;117:734‐749. 28124060 10.1160/TH16-08-0643

[15] Wilson RC , Tambe A , Kidwell MA , Noland CL , Schneider CP , Doudna JA . Dicer‐TRBP complex formation ensures accurate mammalian microRNA biogenesis. Mol Cell. 2015;57:397‐407. 25557550 10.1016/j.molcel.2014.11.030 PMC4320653

[16] Hua JY , He YZ , Xu Y , Jiang XH , Ye W , Pan ZM . Emodin prevents intima thickness via Wnt4/Dvl‐1/beta‐catenin signaling pathway mediated by miR‐126 in balloon‐injured carotid artery rats. Exp Mol Med. 2015;47:e170. 26113441 10.1038/emm.2015.36 PMC4491726

[17] Wang X , Searle AK , Hohmann JD , et al. Dual‐targeted theranostic delivery of miRs arrests abdominal aortic aneurysm development. Mol Ther. 2018;26:1056‐1065. 29525742 10.1016/j.ymthe.2018.02.010 PMC6080135

[18] Morris DR , Biros E , Cronin O , Kuivaniemi H , Golledge J . The association of genetic variants of matrix metalloproteinases with abdominal aortic aneurysm: a systematic review and meta‐analysis. Heart. 2014;100:295‐302. 23813847 10.1136/heartjnl-2013-304129

[19] Petersen E , Gineitis A , Wagberg F , Angquist KA . Activity of matrix metalloproteinase‐2 and ‐9 in abdominal aortic aneurysms. Relation to size and rupture. Eur J Vasc Endovasc Surg. 2000;20:457‐461. 11112465 10.1053/ejvs.2000.1211

[20] Vorkapic E , Folkesson M , Magnell K , Bohlooly YM , Lanne T , Wagsater D . ADAMTS‐1 in abdominal aortic aneurysm. PLoS ONE. 2017;12:e0178729. 28570682 10.1371/journal.pone.0178729 PMC5453572

[21] Gabel G , Northoff BH , Weinzierl I , et al. Molecular fingerprint for terminal abdominal aortic aneurysm disease. J Am Heart Assoc. 2017;6(12):e006798. 29191809 10.1161/JAHA.117.006798 PMC5779007

[22] Lamblin N , Ratajczak P , Hot D , et al. Profile of macrophages in human abdominal aortic aneurysms: a transcriptomic, proteomic, and antibody protein array study. J Proteome Res. 2010;9:3720‐3729. 20513153 10.1021/pr100250s

[23] Gunes MF , Akpinar MB , Comertoglu I , et al. The Investigation of a Disintegrin and Metalloproteinase with ThromboSpondin Motifs (ADAMTS) 1, 5 and 16 in Thoracic Aortic Aneurysms and Dissections. Clin Lab. 2016;62:425‐433. 27156333

[24] Fava M , Barallobre‐Barreiro J , Mayr U , et al. Role of ADAMTS‐5 in Aortic Dilatation and Extracellular Matrix Remodeling. Arterioscler Thromb Vasc Biol. 2018;38:1537‐1548. 29622560 10.1161/ATVBAHA.117.310562 PMC6026471

[25] Tang PC , Coady MA , Lovoulos C , et al. Hyperplastic cellular remodeling of the media in ascending thoracic aortic aneurysms. Circulation. 2005;112:1098‐1105. 16116068 10.1161/CIRCULATIONAHA.104.511717

[26] Campa JS , Greenhalgh RM , Powell JT . Elastin degradation in abdominal aortic aneurysms. Atherosclerosis. 1987;65:13‐21. 3649236 10.1016/0021-9150(87)90003-7

[27] Riches K , Clark E , Helliwell RJ , et al. Progressive development of aberrant smooth muscle cell phenotype in abdominal aortic aneurysm disease. J Vasc Res. 2018;55:35‐46. 29232676 10.1159/000484088

[28] Huang J , Yamashiro Y , Papke CL , et al. Angiotensin‐converting enzyme‐induced activation of local angiotensin signaling is required for ascending aortic aneurysms in fibulin‐4‐deficient mice. Sci Transl Med. 2013;5:183ra58, 1‐11. 10.1126/scitranslmed.3005025 PMC3867808 23636094

[29] Roberts S , Evans H , Wright K , et al. ADAMTS‐4 activity in synovial fluid as a biomarker of inflammation and effusion. Osteoarthritis Cartilage. 2015;23:1622‐1626. 26003949 10.1016/j.joca.2015.05.006 PMC4565717

[30] Tsarouhas A , Soufla G , Katonis P , Pasku D , Vakis A , Spandidos DA . Transcript levels of major MMPs and ADAMTS‐4 in relation to the clinicopathological profile of patients with lumbar disc herniation. Eur Spine J. 2011;20:781‐790. 20857147 10.1007/s00586-010-1573-9 PMC3082673

[31] Lemarchant S , Dunghana H , Pomeshchik Y , et al. Anti‐inflammatory effects of ADAMTS‐4 in a mouse model of ischemic stroke. Glia. 2016;64:1492‐1507. 27301579 10.1002/glia.23017

[32] Longo GM , Xiong W , Greiner TC , Zhao Y , Fiotti N , Baxter BT . Matrix metalloproteinases 2 and 9 work in concert to produce aortic aneurysms. J Clin Invest. 2002;110:625‐632. 12208863 10.1172/JCI15334 PMC151106

[33] Hong‐Brown LQ , Brown CR , Navaratnarajah M , Lang CH . Adamts1 mediates ethanol‐induced alterations in collagen and elastin via a FoxO1‐sestrin3‐AMPK signaling cascade in myocytes. J Cell Biochem. 2015;116:91‐101. 25142777 10.1002/jcb.24945 PMC4718554

[34] Tortorella MD , Liu RQ , Burn T , Newton RC , Arner E . Characterization of human aggrecanase 2 (ADAM‐TS5): substrate specificity studies and comparison with aggrecanase 1 (ADAM‐TS4). Matrix Biol. 2002;21:499‐511. 12392761 10.1016/s0945-053x(02)00069-0

[35] Shiraishi A , Mochizuki S , Miyakoshi A , Kojoh K , Okada Y . Development of human neutralizing antibody to ADAMTS4 (aggrecanase‐1) and ADAMTS5 (aggrecanase‐2). Biochem Biophys Res Commun. 2016;469:62‐69. 26612259 10.1016/j.bbrc.2015.11.072

[36] Majumdar MK , Chockalingam PS , Bhat RA , et al. Immortalized mouse articular cartilage cell lines retain chondrocyte phenotype and respond to both anabolic factor BMP‐2 and pro‐inflammatory factor IL‐1. J Cell Physiol. 2008;215:68‐76. 17960567 10.1002/jcp.21282

[37] Murray PJ , Allen JE , Biswas SK , et al. Macrophage activation and polarization: nomenclature and experimental guidelines. Immunity. 2014;41:14‐20. 25035950 10.1016/j.immuni.2014.06.008 PMC4123412

