# Supplementary figures and images for: MiR‐126a‐5p limits the formation of abdominal aortic aneurysm in mice and decreases ADAMTS‐4 expression
Source: J Cell Mol Med. 2020 May 29;24(14):7896–906. doi: 10.1111/jcmm.15422 (PMC7348185; doi:10.1111/jcmm.15422)

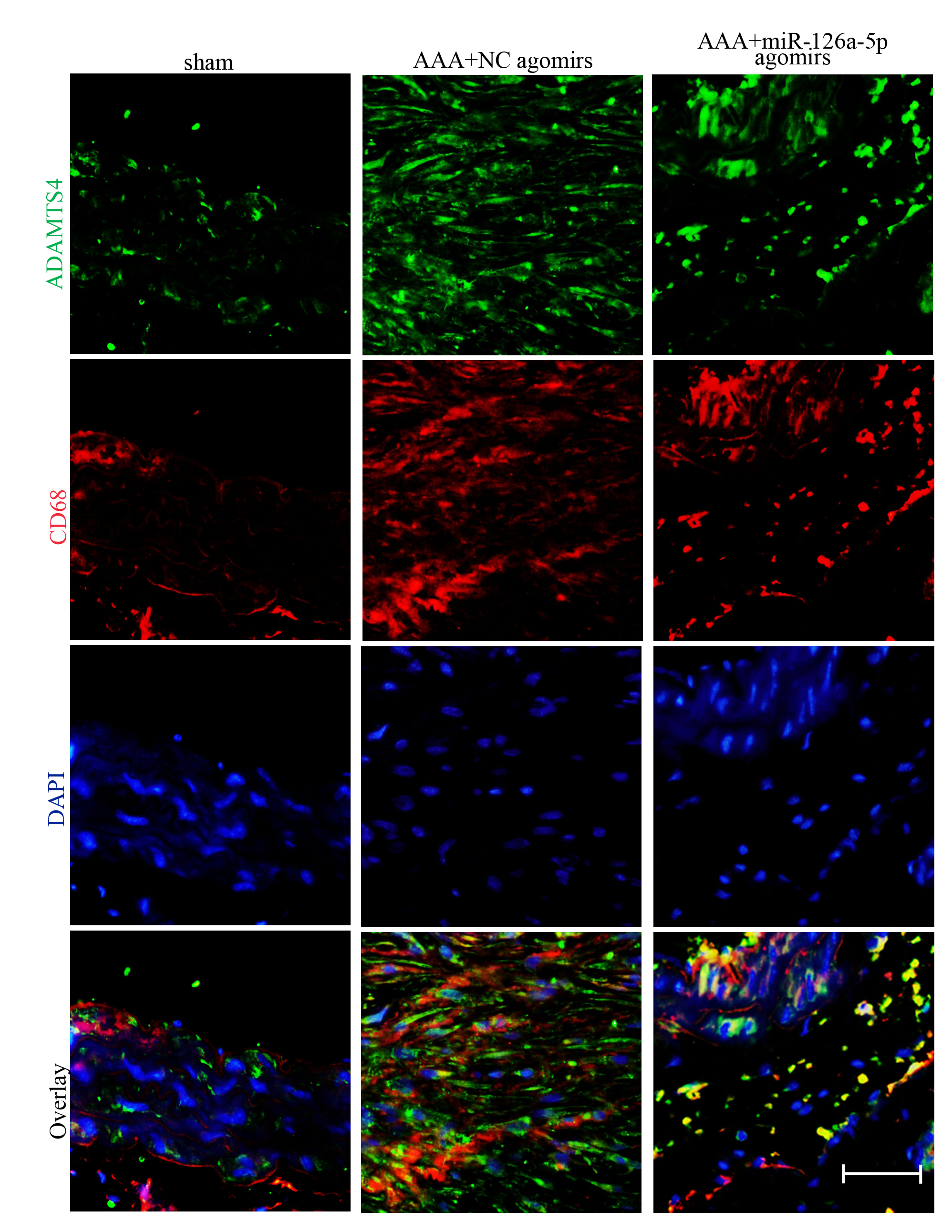

Supplement: Supplementary file 1 — Fig S1 [file JCMM-24-7896-s001.tif]
