# Supplementary material for: MiR‐126a‐5p limits the formation of abdominal aortic aneurysm in mice and decreases ADAMTS‐4 expression
Source: J Cell Mol Med. 2020 May 29;24(14):7896–906. doi: 10.1111/jcmm.15422 (PMC7348185; doi:10.1111/jcmm.15422)
Supplement: Supplementary file 2 — Table S1 [file JCMM-24-7896-s002.doc]

**Table s1** Genes potentially targeted by and negatively expressed (Pearson correlation coefficient < -0.7, and p value < 0.05) with miR-126a-5p in mouse aortic tissues (n = 4 for sham, n = 3 for AAA).

| **miRNA** | **Genes** |
| --- | --- |
| mmu-miR-126a-5p | NWD1 (NACHT and WD repeat domain containing 1) |
|  | BB114814 (Mus musculus expressed sequence BB114814) |
|  | FRMD4A (FERM domain containing 4A) |
|  | EVX2 (Even skipped homeotic gene 2 homolog) |
|  | AHCYL2 (S-adenosylhomocysteine hydrolase-like 2) |
|  | ADAMTS4 (A disintegrin-like and metallopeptidase (reprolysin type) with thrombospondin type 1 motif 4) |
|  | PAX6 (Paired box 6) |
|  | ZFP352 (Zinc finger protein 352) |
|  | CXCL16 (Chemokine (C-X-C motif) ligand 16) |
|  | GM11567 (Mus musculus predicted gene 1156) |
|  | ITPR2 (Inositol 1,4,5-triphosphate receptor 2) |
|  | MCTP2 (clone:F830111H15) |
|  | VMN2R29 (Vomeronasal 2, receptor 29) |
|  | SIS (Sucrase isomaltase (alpha-glucosidase) ) |
|  | VEPH1 (Ventricular zone expressed PH domain-containing 1) |
|  | KCNMA1 (clone:A930011C01) |
|  | TLR8 (Toll-like receptor 8) |
|  | ANPEP (Alanyl (membrane) aminopeptidase) |
|  | GPR34 (G protein-coupled receptor 34) |
|  | ASB11 (Ankyrin repeat and SOCS box-containing 11) |
|  | OLR1 (Oxidized low density lipoprotein (lectin-like) receptor 1) |
|  | SLC24A3 (Solute carrier family 24 (sodium/potassium/calcium exchanger), member 3) |
|  | KLHL29 (Kelch-like 29) |
|  | EFCAB3 (EF-hand calcium binding domain 3) |
|  | GM3740 (clone:D830044C03) |
|  | NRIP1 (Nuclear receptor interacting protein 1 ) |
|  | FBXW23 (F-box and WD-40 domain protein ) |
|  | SYT1 (Synaptotagmin I ) |
|  | BCL6 (B cell leukemia/lymphoma 6) |
|  | MAP3K10 (Mitogen-activated protein kinase kinase kinase 10) |
|  | PTAFR (Platelet-activating factor receptor) |
|  | D430041D05RIK (D430041D05 gene) |
|  | USP29 (Ubiquitin specific peptidase 29) |
|  | CCDC150 (Coiled-coil domain containing 150) |
|  | OLFR1111 (Olfactory receptor 1111) |
|  | CD247 (CD247 antigen) |
