# Supplementary material for: MiR‐126a‐5p limits the formation of abdominal aortic aneurysm in mice and decreases ADAMTS‐4 expression
Source: J Cell Mol Med. 2020 May 29;24(14):7896–906. doi: 10.1111/jcmm.15422 (PMC7348185; doi:10.1111/jcmm.15422)
Supplement: Supplementary file 3 — Supplementary Material [file JCMM-24-7896-s003.docx]

**Fig. s1 ADAMTS-4 increase in infiltrated macrophages within arterial aneurysm is inhibited by miR-126a-5p agomirs**

The protein expression levels of ADAMTS-4 (green) and CD68 (red, a marker for macrophages) were also probed with immunofluorescent assay. Bar, 50 μm.
